# Association of high PM_2.5_ levels with short-term and medium-term lung function recovery in patients with pulmonary lobectomy

**DOI:** 10.3389/fpubh.2022.1022199

**Published:** 2022-10-11

**Authors:** Yi-tong Liu, Yi Xiao, Jian Huang, Hao Hu, Xina Wang, Yueming Chen, Zhiqing Huang, Xiongwen Yang

**Affiliations:** ^1^Department of Thoracic Surgery, Jiangxi Cancer Hospital, Nanchang, China; ^2^School of Ecological Engineering, Guangdong Eco-Engineering Polytechnic, Guangzhou, China; ^3^Guangdong Collaborative Innovation Center of Plant Pest Control and Biological Environmental Health Application Technology, Guangzhou, China; ^4^Guangdong Collaborative Innovation Center of Surveying and Mapping Geographic Information and Forestry Survey Planning, Guangzhou, China; ^5^Department of Cardio-Thoracic Surgery, The Third Affiliated Hospital of Sun Yat-sen University, Guangzhou, China; ^6^Department of Radiation Therapy, General Hospital of Southern Theater Command, Guangzhou, China; ^7^School of Medicine, South China University of Technology, Guangzhou, China

**Keywords:** PM_2.5_, pulmonary lobectomy, lung function, short-term, medium-term

## Abstract

The association between exposure to ambient fine particulate matter with an aerodynamic diameter of ≤ 2.5 μm (PM_2.5_) and short- and medium-term lung function recovery (LFR) in patients undergoing lobectomy remains uncertain. This study investigated the associations between PM_2.5_ concentrations and LFR in adult patients (*n* = 526) who underwent video-assisted thoracoscopic (VATS) lobectomy in Guangzhou, China between January 2018 and June 2021. All patients underwent at least two spirometry tests. Environmental PM_2.5_ concentrations in the same period were collected from the nearest monitoring station. A multiple linear regression (MLR) model was employed to investigate the associations between changes in PM_2.5_ concentrations and LFR in patients who underwent lobectomy after adjusting for potential confounders. We assessed short- and medium-term LFR in patients who underwent lobectomy. The three- and 6-month average PM_2.5_ concentrations in each patient's residential area were divided into regional mild pollution (PM_2.5_ <25 μg/m^3^), moderate pollution (25 μg/m^3^ ≤ PM_2.5_ <35 μg/m^3^), and severe pollution (35 μg/m^3^ ≤ PM_2.5_) periods. The MLR model confirmed that PM_2.5_ was an independent risk factor affecting short-term forced lung capacity (FVC), forced expiratory volume in 1 s (FEV1), and maximum expiratory flow at 50% vital capacity (MEF_50_) recovery (adjusted *P* = 0.041, 0.014, 0.016, respectively). The MLR model confirmed that PM_2.5_ was an independent risk factor affecting medium-term MEF_50_ recovery (adjusted *P* = 0.046). Compared with the moderate and severe pollution periods, the short- and medium-term LFR (FVC, FEV1, MEF_50_) of patients in the mild pollution period were faster and better (*P* < 0.001, *P* < 0.001, *P* < 0.001, *P* = 0.048, *P* = 0.010, *P* = 0.013, respectively). Thus, exposure to high PM_2.5_ levels was associated with significantly reduced speed and degree of short- and medium-term LFR in patients who underwent lobectomy.

## Introduction

Lung cancer is the most lethal malignancy worldwide (1). Patients with surgically resectable lung cancer account for 40% of the total patient population with lung cancer (2). Most patients (70%) with resectable lung cancer undergo pulmonary lobectomy (3). Lobectomy is the surgical removal of the entire lobe of the lung (4), which is still the standard surgical procedure for patients with resectable lung cancer and some benign pulmonary diseases, although some patients with early stage lung cancer now receive sublobectomy (5, 6).

Long-term exposure to air pollution increases the risk of lung cancer and adversely affects lung function (7–12). Particulate matter with an aerodynamic diameter of 2.5 μm or less (PM_2.5_) is one of the most harmful pollutants to lung health. Recently, several studies have shown that air pollution adversely affects lung function in children, adolescents, and adults (10, 13–15). Furthermore, there is growing epidemiological evidence that long-term exposure to air pollution, especially PM_2.5_ and nitrogen dioxide (NO_2_), in healthy people or in people with certain chronic lung diseases, is associated with lower forced lung capacity (FVC) and forced expiratory volume in 1 s (FEV1) (11, 12, 16, 17). The effects of air pollution on lung function may even appear during childhood (15).

Patients who undergo lobectomy lose 7–20% of their baseline lung function (18, 19). Because of intraoperative lung collapse and damage to the thoracic intercostal nerve, short- and medium-term lung function is gradually recovered after surgery (4). The concentration-response curve has shown a continued increase in the death rates associated with higher annual air pollutant concentrations (20), indicating that this is of particular concern in areas with higher air pollution levels, such as China, where PM_2.5_ concentrations often exceed 50 μg/m^3^ (20). A study in Shanghai, China, showed that long-term exposure to high levels of air pollution affects lung function in adults and patients with chronic airway disease (16). Identifying factors that alter the effects of air pollution on the respiratory system is necessary to prevent risks and to help develop interventions to protect vulnerable populations. Therefore, the impact of exposure to air pollution on short- and medium-term lung function recovery (LFR) in patients undergoing pulmonary lobectomy should be examined.

Patients who have undergone pulmonary lobectomy, constitute a large vulnerable group of individuals who will suffer permanent loss of baseline lung function after surgery (4, 6). The degree and speed of lung function recovery in the short- and medium- term are related to the quality of life and perioperative survival of patients (18, 19, 21). Previous studies mainly focused on healthy populations or populations with other diseases and did not explore the population that underwent pulmonary lobectomy (10, 11, 16, 17, 22). There is no evidence that the air environment has an impact of any degree on this group of people.

This is a longitudinal study that investigates the associations of a range of particle metrics with lung function in 526 patients who underwent pulmonary lobectomy in Guangzhou, China, from January 2018 to June 2021, when particulate matter was qualitatively and quantitatively different among periods with different pollution levels.

## Methods

### Study design and population

The current study is a longitudinal study that repeatedly measured each patient's preoperative and postoperative lung function to assess the impact of changes in PM_2.5_ concentrations, on the degree and speed of lung function recovery in the short- and medium-term after pulmonary lobectomy.

**Figure 1:**
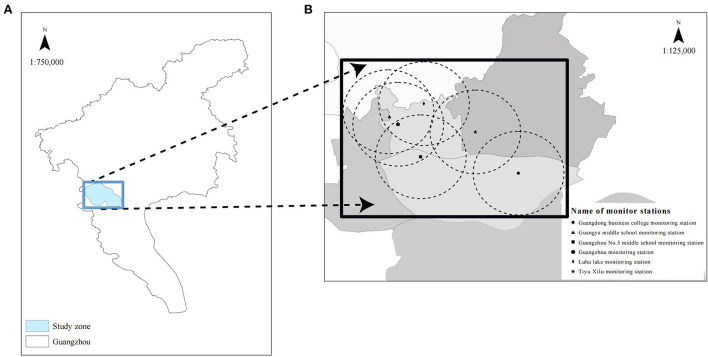
Map of Guangzhou **(A)** showing the location of the study zone (blue area) and that of six monitor stations in the study zone **(B)**. The study zone is in the central city of Guangzhou with a population of over six million.

The data for PM_2.5_, fine particulate matter with a diameter of <10 μm (PM_10_), sulfur dioxide (SO_2_), nitrogen dioxide (NO_2_), carbon monoxide (CO), and ozone (O_3_) and the air quality index (AQI) in the study zone (Figure 1) were obtained from the data of six air monitoring stations located in the central district of Guangzhou city from January 2018 to June 2021. All data including temperature and relative humidity (RH) in the study zone were obtained from the China National Environmental Monitoring Center (http://www.cnemc.cn/en/).

The study zone is located in the central district of Guangzhou, a city in the Pearl River Delta, with a population of over six million. The area is densely populated and small. There is little difference in the annual average fine particulate matter air pollution at each monitoring station, while the monthly average air quality in the Pearl River Delta region fluctuates greatly due to the existence of a winter haze period, making the resident population in this region a natural model to study the effects of fine particulate matter air pollution on lung function in the short- and medium-term (23). Therefore, the geographical location of our study zone is suitable, and the six air monitoring stations included are representative for evaluating the air pollution characteristics of the area.

**Figure 2:**
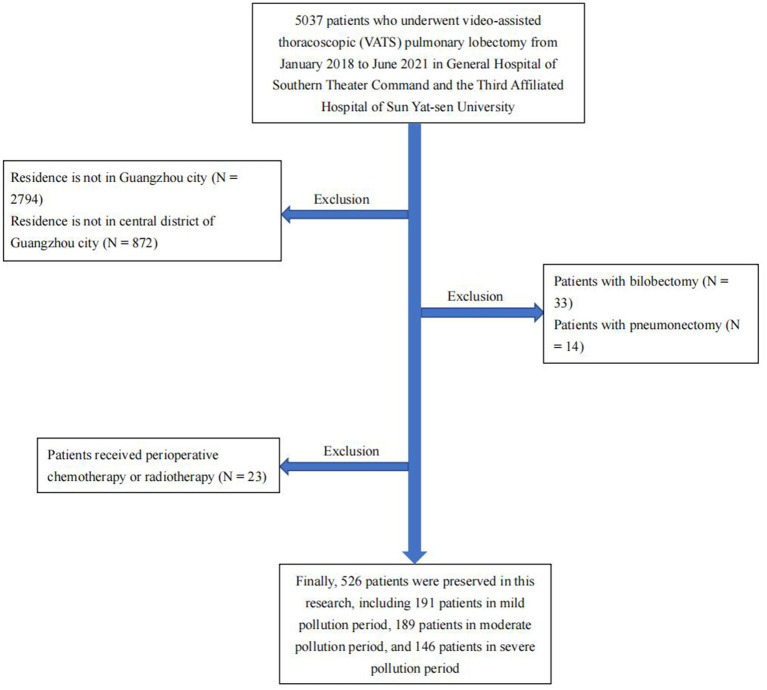
Flow-chart of this study.

This study included patients who underwent video-assisted thoracoscopic (VATS) pulmonary lobectomy in the third affiliated hospital of Sun Yat-Sen University and General Hospital of Southern Theater Command from January 2018 to June 2021 (Figure 2). All patients were registered permanent residents living within four kilometers of the air monitoring stations. To facilitate follow-up statistics, data from the monitoring station with the closest linear distance to the patient's residence were included in follow-up analysis. All patients underwent lung function tests before surgery, and at 3 months after surgery to observe short-term lung function recovery after surgery. Patients with slower lung function recovery in the third month after surgery were re-examined in the sixth month after surgery to observe medium-term lung function recovery after surgery. All patients were instructed on how to perform respiratory rehabilitation training and ensure reasonable nutritional intake before discharge. After discharge, irregular outpatient follow-ups were conducted to provide guidance to patients regarding postoperative rehabilitation. None of the patients had received perioperative chemotherapy or radiotherapy, and their Eastern Cooperative Oncology Group performance status (ECOG PS) was ≤ 2 before surgery and the first month after surgery. None of the patients in this study experienced serious postoperative complications. It is worth noting that all patients undergoing pulmonary lobectomy are routinely advised to quit smoking.

We assessed short-term lung function recovery after lobectomy in all 526 patients. The 3-month average PM_2.5_ concentrations in each patient's residential area were divided into three periods according to the PM_2.5_ concentration levels. In addition, we assessed medium-term lung function recovery in 110 patients with unsatisfactory short-term lung function recovery after surgery and divided the 6-month average PM_2.5_ concentration in each patient's residential area into three periods according to the concentration level. The starting point for calculating the average PM_2.5_ concentration was the time when patients were discharged from the hospital. Meanwhile, we collected environmental PM_2.5_ concentrations from the nearest monitoring station during the same period.

The three periods comprised a regional mild pollution period (PM_2.5_ <25 μg/m^3^), a moderate pollution period (25 μg/m^3^ ≤ PM_2.5_ <35 μg/m^3^), and a severe pollution period (35 μg/m^3^ ≤ PM_2.5_). These three time periods were created for three reasons: first, we performed multiple linear regression (MLR) analysis and confirmed that PM_2.5_ was an independent risk factor affecting postoperative lung function recovery; second, most previous studies were conducted in areas with typical annual PM_2.5_ concentrations of <25 μg/m^3^; third, we took into consideration the secondary standard (35 μg/m^3^) of the national ambient air quality standards in China (GB 3095-2012) (22, 24, 25).

### Lung function test

Lung function tests were conducted by trained and certified technicians using electronic spirometers (ChestGraph HI-101, CHEST Ltd., Tokyo, Japan, or Quark PFT, COSMED Ltd., Rome, Italy). The tests were performed according to the testing protocol of European Respiratory Society/American Thoracic Society standards (ERS/ATS 2005) (26). In addition, we calculated the estimated predicted values of FVC, FEV1, and maximum expiratory flow at 50% vital capacity (MEF_50_) (FVC%pred, FEV1%pred, and MEF_50_%pred, respectively) based on the height, weight, age, and race of each patient (27). Based on preoperative lung function assessments by thoracic surgeons all patients could tolerate lobectomy (4). The following formula was used to evaluate the observed or expected loss of lung function after surgery.


(1) $$\begin{array}{l} Lung\;function\;loss=\;\\ \frac{Preoperative\;lung\;function\;-\;postoperative\;lung\;function\;}{Preoperative\;lung\;function\;}\\ \times 100\% \end{array}$$

### Statistical analysis

Continuous variables are presented as the mean ± standard deviation (SD). We assessed the data for normality using the Shapiro–Wilk test and for homogeneity of variances across period categories using the Bartlett's test for unequal variances (28, 29). Continuous variables in the three periods were assessed using the ANOVA test or Kruskal-Wallis test (30, 31). Categorical data were analyzed using the chi-squared test or Fisher's exact test. The correlation between continuous variables was assessed using Spearman's correlation analysis. Multicollinearity was analyzed by calculating variance inflation factor (VIF). Factors with VIF > 10 were judged as strong collinearity factors and adjusted in the subsequent MLR model.

The association between short-term lung function recovery/loss and exposure to fine particulate matter was assessed using the MLR model (32). Age, sex, BMI, smoking history, chronic obstructive pulmonary disease (COPD), hypertension, diabetes, location of the resected lobe, baseline lung function parameters, meteorological factors (3-month average temperature and relative humidity), and 3-month average concentrations of PM_2.5_, PM_10_, SO_2_, NO_2_, and CO were adjusted in all models. Similarly, the association between medium-term lung function recovery/loss and exposure to fine particulate matter was assessed using the MLR model (32). Age, sex, BMI, smoking history, COPD, hypertension, diabetes, location of the resected lobe, baseline lung function parameters, meteorological factors (6-month average temperature and relative humidity), and 6-month average concentrations of PM_2.5_, PM_10_, SO_2_, NO_2_, and CO were adjusted in all models.

In the analysis of short-term lung function recovery after surgery, the 3-month average PM_2.5_ concentrations in each patient's residential area were divided into three periods according to the concentration level. Baseline data (age, sex, BMI, smoking history, diagnosis, comorbidities, location of the resected lobe, and lung function parameters) were statistically analyzed. The fine particulate matter (PM_2.5_, PM_10_, SO_2_, NO_2_, and CO) concentrations of the three periods were also statistically analyzed. Additionally, meteorological factors (temperature and relative humidity) of the three periods were also statistically analyzed. Similarly, in the analysis of medium-term lung function recovery after surgery, the 6-month average PM_2.5_ concentrations in each patient's residential area were divided into three periods according to the concentration level. Baseline data (age, sex, BMI, smoking history, diagnosis, comorbidities, location of the resected lobe, and lung function parameters) were statistically analyzed. The fine particulate matter (PM_2.5_, PM_10_, SO_2_, NO_2_, and CO) concentrations of the three periods were also statistically analyzed. Additionally, meteorological factors (temperature and relative humidity) of the three periods were also statistically analyzed.

All statistical analyses were performed using R (version 3.6.1, R Foundation for Statistical Computing, http://cran.r-project.org/). A *P-*value of < 0.05 was considered statistically significant for all results.

## Results

### Study population and patient characteristics

In total, 526 patients were included in this study (Table 1). All patients underwent at least two lung function tests, whereas 110 patients underwent three lung function tests. Of the 526 patients who were assessed for short-term lung function recovery after surgery, 191 recovered in the mild pollution period, 189 in the moderate pollution period, and 146 in the severe pollution period. Of the 110 patients who were assessed for medium lung function recovery after surgery, 23 recovered in the mild pollution period, 63 in the moderate pollution period, and 24 in the severe pollution period. In the short-term lung function recovery group, there were no statistically significant differences in baseline data (age, sex, BMI, smoking history, diagnosis, comorbidities, location of the resected lobe, and lung function parameters) among the three periods. In the medium-term lung function recovery group, there were no statistically significant differences in baseline data (age, sex, BMI, diagnosis, comorbidities, location of resected lobe, and lung function parameters) among the three periods, but there were statistically significant differences in smoking history among patients at the three time points (*P* = 0.041).

**Table 1:** Demographic characteristic of patients with pulmonary lobectomy during three periods.

**Variables**	**All patients**	**Mild pollution period**	**Moderate pollution period**	**Severe pollution period**	***P-*value**
		**PM_2.5_ <25 ug/m^3^**	**25 ug/m^3^ ≤ PM_2.5_ <35 ug/m^3^**	**35 ug/m^3^ ≤ PM_2.5_**	
**Short-term (3 months)**	***N =*** **526**	**Patients (*****N =*** **191)**	**Patients (*****N =*** **189)**	**Patients (*****N =*** **146)**	
**Age** (Mean ± SD) year	61.38 ± 10.72	61.05 ± 11.16	60.81 ± 10.25	62.53 ± 10.69	0.220
**Sex**					0.523
Male	292	100	107	85	
Female	234	91	82	61	
**BMI** (Mean ± SD) Kg/m^2^	22.99 ± 3.05	22.17 ± 3.03	23.1 ± 3.05	23.2 ± 3.08	0.200
**Smoking history**					0.089
Never	304	102	121	81	
Former or current	222	89	68	65	
**Diagnosis**					0.867
Malignant	463	170	165	128	
Benign	63	21	24	18	
**Comorbidity**					
COPD	86	31	36	19	0.334
Hypertension	84	28	30	26	0.736
Diabetes	73	25	29	19	0.767
**Resected_lobe**					0.448
Left lower	93	34	32	27	
Left upper	143	48	54	41	
Right lower	88	35	37	16	
Right middle	39	18	11	10	
Right upper	163	56	55	52	
**Lung function**					
FVC (Mean ± SD) L	2.92 ± 0.66	2.92 ± 0.62	2.88 ± 0.65	2.97 ± 0.72	0.650
FEV1 (Mean ± SD) L	2.38 ± 0.52	2.4 ± 0.55	2.34 ± 0.51	2.41 ± 0.56	0.500
MEF50 (Mean ± SD) L/S	3.1 ± 0.97	3.15 ± 1.03	3.06 ± 0.97	3.12 ± 0.87	0.470
FVC %predicted (%)	91.49 ± 13.36	91.17 ± 13.36	91.53 ± 13.69	91.86 ± 12.88	0.820
FEV1 %predicted (%)	92.44 ± 12.90	92.48 ± 12.97	91.81 ± 13.47	93.2 ± 12.07	0.620
MEF_50_ %predicted (%)	82.65 ± 23.12	82.77 ± 23.47	81.64 ± 23.88	83.81 ± 21.73	0.670
**Medium-term (6 months)**	***N =*** **110**	***N =*** **23**	***N =*** **63**	***N =*** **24**	
**Age** (Mean ± SD) year	62.51 ± 10.12	59.13 ± 10.11	62.76 ± 9.85	65.08± 10.36	0.130
**Sex**					0.160
Male	74	14	40	20	
Female	36	9	23	4	
**BMI** (Mean ± SD) Kg/m^2^	22.89 ± 2.88	22.96 ± 3.10	22.71 ± 2.75	23.29 ± 3.07	0.700
**Smoking history**					0.041
Never	70	16	44	10	
Former or current	40	7	19	14	
**Diagnosis**					0.720
Malignant	101	23	58	21	
Benign	9	1	5	3	
**Comorbidity**					
COPD	11	3	6	2	0.830
Hypertension	14	2	10	2	0.580
Diabetes	10	1	7	2	0.820
**Resected_lobe**					0.160
Left lower	14	1	9	4	
Left upper	87	21	50	16	
Right lower	7	1	3	4	
Right upper	2	0	1	0	
**Lung function**					
FVC (Mean ± SD) L	2.86 ± 0.72	2.73 ± 0.73	2.88 ± 0.73	2.95 ± 0.75	0.600
FEV1 (Mean ± SD) L	2.34 ± 0.54	2.33 ± 0.60	2.37 ± 0.53	2.27 ± 0.55	0.600
MEF50 (Mean ± SD) L/S	3.12 ± 0.89	3.29 ± 0.96	3.17 ± 0.89	2.83 ± 0.78	0.160
FVC %predicted (%)	93.17 ± 13.20	89.52 ± 12.86	93.71 ± 13.44	95.24 ± 12.72	0.300
FEV1 %predicted (%)	94.52 ± 11.98	93.38 ± 13.06	95.45 ± 12.45	93.16 ± 9.63	0.640
MEF_50_ %predicted (%)	84.52 ± 22.64	83.66 ± 24.19	87.42 ± 23.46	78.2 ± 17.92	0.230

SD, Standard deviation; COPD, Chronic obstructive pulmonary disease; BMI, Body mass index; FVC, Forced vital capacity; FEV1, Forced expiratory volume in 1 s; MEF_50_, Maximum expiratory flow at 50% vital capacity.

### Air pollution exposure and meteorological factors

**Figure 3:**
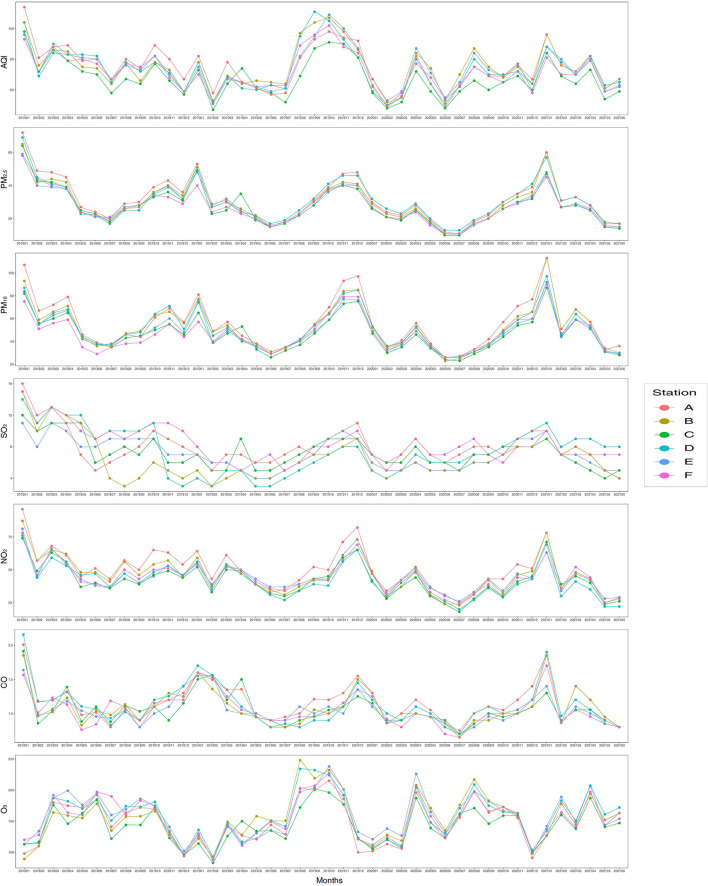
Average monthly air quality index (AQI) and exposure to PM_2.5_, PM_10_, SO_2_, NO_2_, CO, and O_3_ in the study area from January 2018 to June 2021. Station A: Guangya middle school monitoring station; Station B: Guangzhou monitoring station; Station C: Luhu lake monitoring station; Station D: Guangzhou No. 5 middle school monitoring station; Station E: Guangdong business college monitoring station; Station F: Tiyu Xilu monitoring station. PM_2.5_, fine particulate matter with a diameter of < 2.5 μm; PM_10_, fine particulate matter with a diameter of < 10 μm; SO_2_, sulfur dioxide; NO_2_, nitrogen dioxide; CO, carbon monoxide, O_3_, ozone.

From January 2018 to June 2022, except for the monthly average concentration of SO_2_, the trends of the monthly average AQI and monthly average concentrations of PM_2.5_, PM_10_, NO_2_, CO, and O_3_ of the six air monitoring stations in our study zone were similar (Figure 3). In addition, the monthly average AQI and monthly average concentrations of PM_2.5_, PM_10_, NO_2_, CO, and O_3_ fluctuated significantly in the study zone (Figure 3). From January 2018 to June 2022, the average concentrations of PM_2.5_, PM_10_, SO_2_, NO_2_, CO, and O_3_ in the study zone were 29.18 μg/m^3^, 51.05 μg/m^3^, 7.59 μg/m^3^, 43.95 μg/m^3^, 1.2 mg/m^3^, and 167.18 μg/m^3^, respectively. The average AQI was 72.59 in the study zone (Table 2). In addition, the average temperature and relative humidity in study zone were 22.96°C and 79.79%, respectively (Table 2). In general, the average AQI and average concentrations of PM_2.5_, PM_10_, SO_2_, NO_2_, and CO were the highest during the severe pollution period, followed by those in the moderate and mild pollution periods. In contrast, the average concentrations of O_3_ were the highest in the mild pollution period, followed by those in the moderate and severe pollution periods (Table 2 and Supplemenatry material).

**Table 2:** Descriptive statistics of pollutant concentrations and meteorological factors during three periods.

**Variables (Mean ±SD)**	All period*	**Mild pollution period**	**Moderate pollution period**	**Severe pollution period**	***P*-value**
**Short-term (3 months)**		**PM**_**2.5**_ **<25 ug/m**^**3**^	**25 ug/m**^**3**^ **≤PM**_**2.5**_ **<35 ug/m**^**3**^	**35 ug/m**^**3**^ **≤PM**_**2.5**_	
AQI	72.59 ± 5.73	67.73 ± 6.43	72.22 ± 10.78	73.65 ± 11.45	<0.0001
PM_2.5_ (ug/m^3^)	29.18 ± 4.63	19.75 ± 3.21	29.67 ± 2.76	39.01 ± 3.6	<0.0001
PM_10_ (ug/m^3^)	51.05 ± 6.11	36.25 ± 4.58	50.4 ± 6.26	65.47 ± 6.91	<0.0001
SO_2_ (ug/m^3^)	7.59 ± 1.44	6.51 ± 1.67	7.6 ± 2.03	8.41 ± 2.43	<0.0001
NO_2_ (ug/m^3^)	43.95 ± 6.79	36.69 ± 5.72	44.94 ± 5.05	51.94 ± 5.71	<0.0001
CO (mg/m^3^)	1.2 ± 0.12	0.916 ± 0.08	1.11 ± 0.08	1.24 ± 0.13	<0.0001
O_3_ (ug/m^3^)	167.18 ± 10.85	158.85 ± 19.05	151.32 ± 31.60	146.71 ± 25.47	<0.0001
Temperature (°C)	22.96 ± 5.16	23.16 ± 4.61	22.70 ± 3.98	22.82 ± 2.36	0.550
Relative humidity (%)	79.79 ± 6.86	79.90 ± 5.93	79.59 ± 5.02	79.44 ± 6.33	0.840
**Medium-term (6 months)**					
AQI		65.65 ± 4.15	71.02 ± 6.25	74.42 ± 5.99	<0.0001
PM_2.5_ (ug/m^3^)		21 ± 2	29.97 ± 2.15	36.08 ± 1.18	<0.0001
PM_10_ (ug/m^3^)		38.13 ± 3.35	52.78 ± 5.90	60.25 ± 5.64	<0.0001
SO_2_ (ug/m^3^)		6.43 ± 1.04	7.54 ± 1.56	7.38 ± 2.39	0.009
NO_2_ (ug/m^3^)		36.61 ± 4.55	45.89 ± 3.40	48.33 ± 4.72	<0.0001
CO (mg/m^3^)		0.955 ± 0.05	1.12 ± 0.09	1.214 ± 0.10	<0.0001
O_3_ (ug/m^3^)		152.61 ± 14.29	147.16 ± 17.11	146.25 ± 16.74	0.330
Temperature (°C)		22.20 ± 2.62	23.38 ± 2.49	21.93 ± 3.06	0.820
Relative humidity (%)		75.52 ± 6.92	77.99 ± 6.42	78.83 ± 5.67	0.900

SD, Standard deviation.

^*^Variables (Mean ± SD) of each indicator in study zone from 2018 to June 2021.

During the mild pollution period, the 3-month average concentration of PM_2.5_ was the lowest at 12 μg/m^3^. During the severe pollution period, the 3-month average concentration of PM_2.5_ was the highest at 52 μg/m^3^. In addition, during the mild pollution period, the 6-month average concentration of PM_2.5_ was the lowest at 15 μg/m^3^. During the severe pollution period, the 6-month average concentration of PM_2.5_ was the highest at 47 μg/m^3^. Hence, the AQI and fine particulate matter concentrations of the three periods differed greatly (Figure S1).

### Associations of PM_2.5_ and its constituents with lung function

Table 3 shows that the MLR model confirmed that the concentration of PM_2.5_ was an independent risk factor affecting short-term FVC, FEV1, and MEF_50_ recovery (adjusted *P* = 0.041, 0.014, 0.016, respectively). Table 4 shows that the MLR model confirmed that PM_2.5_ was an independent risk factor affecting medium-term MEF_50_ recovery (adjusted *P* = 0.003).

**Table 3:** Impact factors of short-term lung function loss after pulmonary lobectomy.

**Variables**	**FVC loss**		**FEV1 loss**		**MEF**_**50**_ **loss**	
	**Unadjusted *P-*value**	Adjusted *P-*value*	**Unadjusted *P-*value**	**Adjusted *P-*value**	**Unadjusted *P-*value**	**Adjusted *P-*value**
**Age**	0.024	0.035	0.069	0.124	0.154	0.314
**Sex**						
M	0.230	0.052	0.289	0.033	0.111	<0.001
F	Ref	Ref	Ref	Ref	Ref	Ref
**BMI**	0.882	0.816	0.747	0.75	0.766	0.873
**Smoking history**						
Y	0.044	0.003	0.013	0.001	0.015	<0.001
N	Ref	Ref	Ref	Ref	Ref	Ref
**COPD**						
Y	0.145	0.035	0.019	0.106	0.002	<0.001
N	Ref	Ref	Ref	Ref	Ref	Ref
**Hypertension**						
Y	0.646	0.029	0.467	0.021	0.527	0.020
N	Ref	Ref	Ref	Ref	Ref	Ref
**Diabetes**						
Y	0.775	0.144	0.148	0.001	0.094	<0.001
N	Ref	Ref	Ref	Ref	Ref	Ref
**Resected lobe**						
Left lower	Ref	Ref	Ref	Ref	Ref	Ref
Left upper	<0.001	<0.001	<0.001	<0.001	<0.001	<0.001
Right lower	0.134	0.223	0.084	0.110	0.954	0.734
Right middle	<0.001	<0.001	<0.001	<0.001	<0.001	<0.001
Right upper	<0.001	<0.001	<0.001	<0.001	<0.001	<0.001
**Baseline FVC**	0.050	0.011	0.062	0.866	0.041	0.435
**Baseline FEV1**	0.310	0.084	0.020	0.654	0.017	0.669
**Baseline MEF**_**50**_	0.803	0.428	0.133	0.811	0.113	0.629
**PM**_**2.5**_ **_3M**	<0.001	0.041	<0.001	0.014	<0.001	0.016
**PM**_**10**_ **_3M**	<0.001	0.472	<0.001	0.319	<0.001	0.156
**SO**_**2**_ **_3M**	0.072	0.497	0.154	0.121	0.081	0.039
**NO**_**2**_ **_3M**	<0.001	0.420	<0.001	0.404	<0.001	0.725
**CO_3M**	<0.001	0.037	<0.001	0.598	<0.001	0.537
**Temperature**	0.895	0.102	0.238	0.918	0.203	0.748
**Relative humidity**	0.225	0.103	0.096	0.187	0.18	0.528

^*^Adjusted P-values were obtained by adjusting for confounding factors (Age, sex, BMI, smoking history, COPD, hypertension, diabetes, location of resected lobe, baseline lung function parameters, and 3-month average concentration of PM_2.5_, PM_10_, NO_2_, and CO) through multiple linear regression models.

FVC, Forced vital capacity; FEV1, Forced expiratory volume in 1 s; MEF_50_, Maximum expiratory flow at 50% vital capacity; BMI, Body mass index; COPD, Chronic obstructive pulmonary disease; PM_2.5_, Fine particulate matter with a diameter < 2.5 μm; PM_10_, Fine particulate matter with a diameter < 10 μm; SO_2_, Sulfur dioxide; NO_2_, Nitrogen dioxide; CO, Carbon monoxide; 3M, 3-month average.

**Table 4:** Impact factors of medium-term lung function loss after pulmonary lobectomy.

**Variables**	**FVC loss**		**FEV1 loss**		**MEF**_**50**_ **loss**	
	**Unadjusted *P-*value**	Adjusted *P-*value*	**Unadjusted *P-*value**	**Adjusted *P-*value**	**Unadjusted *P-*value**	**Adjusted *P-*value**
**Age**	0.684	0.545	0.856	0.027	0.281	0.414
**Sex**						
M	0.484	0.577	0.629	0.602	0.656	0.740
F	Ref	Ref	Ref	Ref	Ref	Ref
**BMI**	0.761	0.263	0.686	0.204	0.948	0.729
**Smoking history**						
Y	0.136	0.561	0.087	0.344	0.565	0.397
N	Ref	Ref	Ref	Ref	Ref	Ref
**COPD**						
Y	0.029	0.111	0.357	0.543	0.084	0.355
N	Ref	Ref	Ref	Ref	Ref	Ref
**Hypertension**						
Y	0.890	0.101	0.697	0.270	0.914	0.356
N	Ref	Ref	Ref	Ref	Ref	Ref
**Diabetes**						
Y	0.428	0.377	0.009	0.083	0.337	0.851
N	Ref	Ref	Ref	Ref	Ref	Ref
**Resected lobe**						
Left lower	Ref	Ref	Ref	Ref	Ref	Ref
Left upper	<0.001	<0.001	<0.001	<0.001	0.02	0.003
Right lower	0.717	0.529	0.204	0.130	0.111	0.060
Right upper	0.140	0.047	0.019	0.008	0.495	0.342
**Baseline FVC**	0.239	0.397	0.518	0.660	0.094	0.782
**Baseline FEV1**	0.071	0.461	0.168	0.877	0.125	0.845
**Baseline MEF**_**50**_	0.633	0.209	0.572	0.602	0.621	0.840
**PM**_**2.5**_ **_6M**	0.160	0.118	0.288	0.361	0.003	0.046
**PM**_**10**_ **_6M**	0.199	0.677	0.283	0.861	0.006	0.854
**SO**_**2**_ **_6M**	0.746	0.134	0.286	0.573	0.207	0.873
**NO**_**2**_ **_6M**	0.085	0.545	0.231	0.751	0.090	0.337
**CO_6M**	0.260	0.837	0.374	0.154	0.111	0.886
**Temperature**	0.464	0.606	0.989	0.557	0.739	0.694
**Relative humidity**	0.457	0.320	0.389	0.383	0.936	0.624

^*^Adjusted P-values were obtained by adjusting for confounding factors (Age, sex, height, weight, BMI, smoking history, COPD, hypertension, diabetes, location of resected lobe, baseline lung function parameters, 6-month average concentration of PM_2.5_, PM_10_, NO_2_, and CO) through multiple linear regression models.

FVC, Forced vital capacity; FEV1, Forced expiratory volume in 1 s; MEF_50_, Maximum expiratory flow at 50% vital capacity; BMI, Body mass index; COPD, Chronic obstructive pulmonary disease; PM_2.5_, Fine particulate matter with a diameter < 2.5 μm; PM_10_, Fine particulate matter with a diameter < 10 μm; SO_2_, Sulfur dioxide; NO_2_, Nitrogen dioxide; CO, Carbon monoxide; 6M, 6-month average.

**Figure 4:**
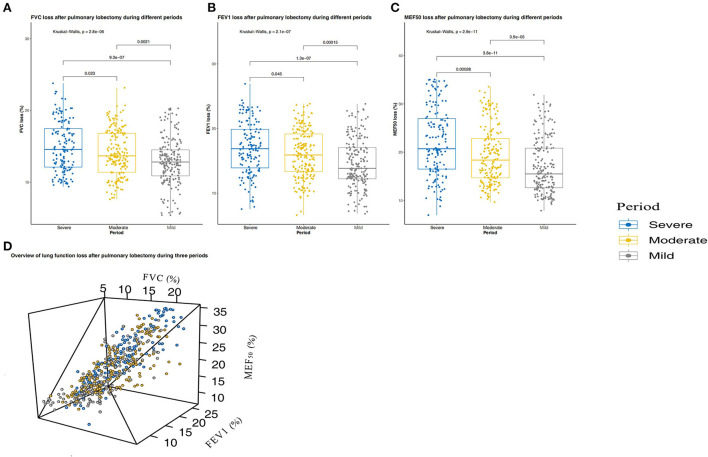
Short-term (3 months) lung function changes in patients with pulmonary lobectomy during the three periods. **(A)** Forced vital capacity (FVC) change after pulmonary lobectomy during the three periods. **(B)** Forced expiratory volume in 1 s (FEV1) change after pulmonary lobectomy during the three periods. **(C)** Maximum expiratory flow at 50% vital capacity (MEF_50_) change after pulmonary lobectomy during the three periods. **(D)** Overview of lung function loss after pulmonary lobectomy during the three periods. The three periods are periods with mild, moderate, and severe pollution.

**Figure 5:**
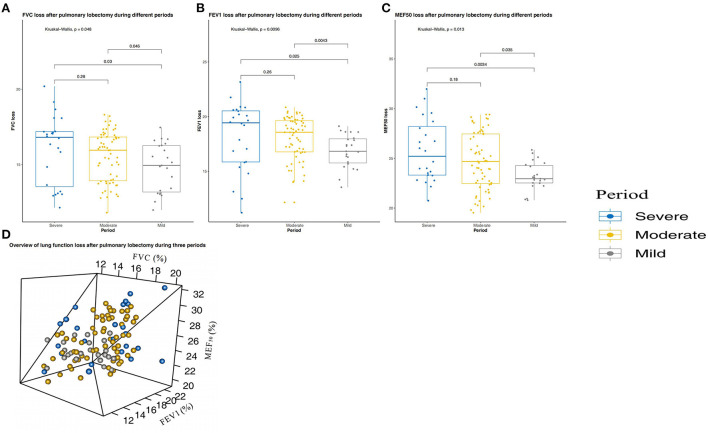
Medium-term (6 months) lung function changes in patients with pulmonary lobectomy during the three periods. **(A)** Forced vital capacity (FVC) change after pulmonary lobectomy during the three periods. **(B)** Forced expiratory volume in 1 s (FEV1) change after pulmonary lobectomy during the three periods. **(C)** Maximum expiratory flow at 50% vital capacity (MEF_50_) change after pulmonary lobectomy during the three periods. **(D)** Overview of lung function loss after pulmonary lobectomy during the three periods. The three periods are periods with mild, moderate, and severe pollution.

The largest difference between the three periods was in the average concentration of PM_2.5_ (Figure S1). Figure 4 shows only a small loss of short-term lung function after surgery in patients who underwent lobectomy during the mild pollution period, indicating that patients had faster and better lung function (FVC, FEV1, and MEF_50_) recovery after surgery during this period (*P* < 0.001, *P* < 0.001, *P* < 0.001, respectively). In addition, we assessed the medium-term recovery of lung function in patients with unsatisfactory short-term recovery of lung function after surgery. Figure 5 shows that medium-term lung function (FVC, FEV1, and MEF_50_) recovery in patients during the mild pollution period was better than that in patients during the moderate and severe pollution periods (*P* = 0.048, *P* = 0.010, *P* = 0.013, respectively). However, the medium-term recovery of lung function in patients was not statistically different during the moderate and severe pollution periods.

## Discussion

Ambient air pollution increases morbidity and mortality associated with respiratory diseases (20, 25). Long-term exposure to ambient air pollution can impair lung function in people of all ages (14–16). In this longitudinal study of patients with pulmonary lobectomy from Guangzhou, China, we found that the three- and 6-month average concentrations of PM_2.5_ were significantly associated with higher FVC loss, FEV1 loss, and MEF_50_ loss, indicating that air pollution-related lung function was impaired in the short- and medium-term.

In contrast to most studies, a special population of patients with pulmonary lobectomy was included in the current study to explore the relationship between lung function and PM_2.5_ concentration. Given the high incidence of lung cancer, a large patient population has the opportunity to undergo pulmonary lobectomy. Previous studies on the factors influencing lung function recovery after pulmonary lobectomy mostly focused on medical perspectives, such as analysis of physical fitness and postoperative complications (18, 19, 21). However, it is unclear whether the external environment influences lung function recovery and to what extent. Few clinicians have paid attention to this critical issue in clinical practice. In fact, many previous studies have confirmed that even short-term exposure to PM_2.5_ can impair lung function in healthy people and patients with COPD (33–36). Furthermore, a few previous studies have confirmed that long-term exposure to PM_2.5_ could impair small airway function (12, 37, 38). The MEF_50_ is an important indicator of small airway function. In this study, we observed that exposure to ambient PM_2.5_ generally had a stronger association with MEF_50_ than with FVC and EFV1. This finding suggests that short- and medium-term exposure to PM_2.5_ may have more serious effects on small airway function than on large airway function in this vulnerable population.

The degree of lung function loss or recovery in patients undergoing pulmonary lobectomy is related to the location of the resected lobe (18, 19). Patients who underwent right middle lobectomy lost approximately 7% of their FVC and 12% of their FEV1 (18). However, patients with left upper lobectomy lost approximately 17% of their FVC and 18% of their FEV1 (18). Our MLR model also showed that the location of the resected lobe was an independent risk factor for loss of lung function (Tables 3, 4). In addition, short-term lung function recovery was worse in current/former smokers and in men. Previous studies have shown negative associations between FVC and FVC%pred in never-smokers and women (16). Furthermore, comorbidities can affect the recovery of short-term postoperative lung function, which may be due to the relatively poor physical fitness of these patients (18). Due to the degree of postoperative pain and the possible effects of respiratory rehabilitation training on lung function recovery, a conventional outpatient follow-up was conducted in the first month after surgery to evaluate patient recovery. Most patients can undergo respiratory rehabilitation training. Patients with poor recovery were irregularly followed up to assist them with respiratory rehabilitation training. Finally, chest CT reexamination of all patients 3 months after surgery indicated good pulmonary re-expansion.

Different from other regions, the monthly average concentration of PM_2.5_ in the Pearl River Delta region fluctuates greatly due to the existence of a winter haze period (23). In our study zone, the 3-month average concentration of PM_2.5_ was 12 μg/m^3^ at the lowest level and 52 μg/m^3^ at the highest level, and the 6-month average concentration of PM_2.5_ was 15 μg/m^3^ at the lowest level and 47 μg/m^3^ at the highest level. In addition, several studies used a spatiotemporal model based on high spatial resolution satellite data to estimate the PM_2.5_ exposure for each participant's address, which were more precise and reliable than studies based on data from monitoring stations. Furthermore, the regions investigated in these studies are large and geographically complex, the study span is long, and a high-precision spatiotemporal model is necessary. However, our study zone is in the central district of Guangzhou city, with a narrow geographical area and large population. This is a typical metropolitan residential area without large factories and forests, and its geographical environment is relatively simple. Therefore, data from air monitoring stations in the area are appropriate. In this area, there is no significant spatial difference in the monthly average PM_2.5_ exposure level, but there is a significant time difference, which becomes a natural model of air environment change in the short- and medium-term.

This study had several important strengths. First, it further supports the first evidence of negative associations between short- and medium-term PM_2.5_ exposure and lung function in areas with low to high levels of air pollution in a special population. Second, all patients underwent at least two lung function tests, which enabled longitudinal analysis. Third, unlike other studies based on healthy participants, our study population comprised postoperative patients and multiple follow-up visits with detailed data. In the MLR model, we adjusted for potential confounders and reduced the bias. Finally, to the best of our knowledge, this study is the first to investigate the relationship between PM_2.5_ concentration and the speed and degree of short- and medium-term lung function recovery in patients with pulmonary lobectomy.

Our study had several limitations. First, not every patient underwent lung function tests at the sixth month after surgery, and only 20% of patients were included in the assessment of the relationship between PM_2.5_ concentration and medium-term lung function recovery. Even in the case of poor short-term recovery of lung function, the lung function of patients during mild pollution periods was significantly recovered compared to that of patients during moderate and severe pollution periods. Although there was no statistical difference in lung function recovery/loss between patients during moderate pollution periods and severe pollution periods in the medium-term lung function assessment, we still observed a different trend between the two periods, which may become statistically significant in larger populations. Second, although we adjusted for many personal and environmental factors, there is still uncertainty in estimating individual exposure due to unmeasured contaminant levels at the micro-environmental level. In addition, postoperative patients may be in an indoor environment for a relatively long time, and the effect of the air pollution level on the degree and speed of lung function recovery in patients remains to be further studied. Third, when evaluating medium-term lung function recovery, we noticed that the short-term lung function evaluations of some patients were conducted during mild or severe pollution periods, whereas the medium-term lung function evaluations of these patients were conducted during moderate pollution periods. Owing to the small number of patients included in the medium-term lung function evaluation, it is difficult to obtain robust statistical analysis results regarding differences in lung function recovery between patients in mild and moderate pollution periods and between patients in severe and moderate pollution periods. Finally, the lung function recovery of patients after lobectomy in areas with low PM_2.5_ concentration in the Pearl River Delta was not included in our study. In our study zone, the 3-month average concentration of PM_2.5_ was 19.75 μg/m^3^, and the 6-month average concentration of PM_2.5_ was 21 μg/m^3^, even in the mild pollution period. This is far higher than the concentration of PM_2.5_ recommended by the World Health Organization Global Air Quality Guidelines (WHO AQG) (39). We will further include the data of short- and medium-term lung function recovery in patients with pulmonary lobectomy in areas with low PM_2.5_ concentration, to explore the impact of low PM_2.5_ exposure on this particular population.

## Conclusions

Our findings suggest that exposure to high levels of ambient PM_2.5_ are associated with significantly reduced speed and degree of short- and medium-term lung function recovery in Chinese patients undergoing pulmonary lobectomy. The effects were modified by age, sex, BMI, smoking history, COPD, hypertension, diabetes, location of the resected lobe, baseline lung function parameters, and monthly or monthly average concentrations of PM_10_, SO_2_, NO_2_, and CO, allowing for the identification of related risk factors. Further research is needed to replicate these findings in populations exposed to a wider range of pollutant concentrations, and to make recommendations for reducing particulate matter air pollution exposure during recovery in this vulnerable population. However, a small indoor air purifier may be helpful for lung function recovery in patients undergoing pulmonary lobectomy.

## Data availability statement

The raw data supporting the conclusions of this article will be made available by the authors, without undue reservation.

## Ethics statement

The studies involving human participants were reviewed and approved by the Ethics Committee of Medical Scientific Research Foundation of Guangdong Province, China (Ethics Clearance:2021[073]). The patients/participants provided their written informed consent to participate in this study.

## Author contributions

Conception and design: XWY, JH, and YX. Development of methodology and administrative, technical, or material support: XWY, YTL, JH, YX, XNW, YMC, and ZQH. Acquisition of data: XY, YTL, YX, and HH. Analysis and interpretation of data: XWY, YTL, JH, YX, XNW, and HH. Writing, review, and revision of the manuscript: XWY, YTL, JH, YX, XNW, YMC, ZQH, and HH. Study supervision: XY, YTL, YX, JH, and XNW. Other (algorithm and software development): XY, YTL, and XNW. All authors contributed to the article and approved the submitted version.

## Funding

This research was supported by the National Natural Science Foundation of China (41801074 and 41871085), Medical Scientific Research Foundation of Guangdong Province, China (No. A2022142), Research and Practice of Education and Teaching Reform in Guangdong Province (GDGZ19Y066), Innovative Project of Ordinary University in Guangdong Province (2019GKTSCX067), and Youth Innovative Talents Project of Guangdong Provincial Department of Education (2018GkQNCX115).

## Conflict of interest

The authors declare that the research was conducted in the absence of any commercial or financial relationships that could be construed as a potential conflict of interest.

## Publisher's note

All claims expressed in this article are solely those of the authors and do not necessarily represent those of their affiliated organizations, or those of the publisher, the editors and the reviewers. Any product that may be evaluated in this article, or claim that may be made by its manufacturer, is not guaranteed or endorsed by the publisher.

## Abbreviations

LFR: lung function recovery
PM_2.5_: particulate matter with an aerodynamic diameter of ≤ 2.5 μm
VATS: video-assisted thoracoscopic
MLR: multiple linear regression
FVC: forced lung capacity
FEV1, forced expiratory volume in 1 s, MEF_50_: maximum expiratory flow at 50% vital capacity
AQI: average monthly air quality index
PM_10_: fine particulate matter with a diameter of <10 μm
SO_2_: sulfur dioxide
NO_2_: nitrogen dioxide
CO: carbon monoxide
O_3_: ozone
COPD: chronic obstructive pulmonary disease.
