# Supplementary figures and images for: Association of high PM2.5 levels with short-term and medium-term lung function recovery in patients with pulmonary lobectomy
Source: Front Public Health. 2022 Oct 11;10:1022199. doi: 10.3389/fpubh.2022.1022199 (PMC9593074; doi:10.3389/fpubh.2022.1022199)

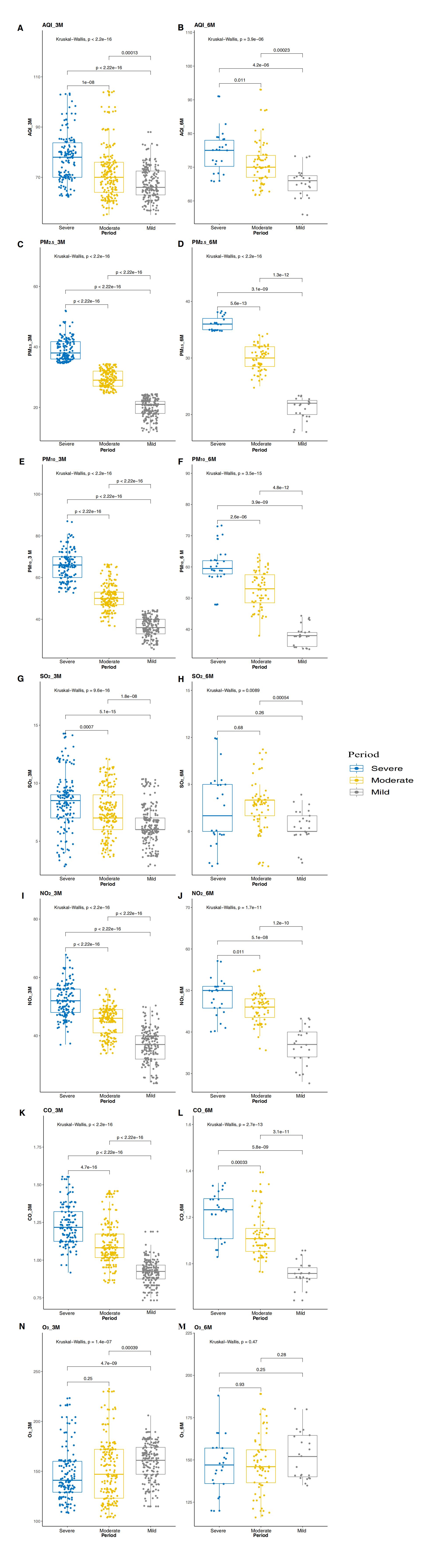

Supplement: Supplementary file 1 [file Image_1.JPEG]
